# Protective function of the voltage-gated potassium channel Kv11.3 in a mouse model of cardiac ischemia/reperfusion injury

**DOI:** 10.1371/journal.pone.0323428

**Published:** 2025-05-07

**Authors:** Hayato Sasaki, Kazuki Otake, Kazuki Takeda, Karin Tesaki, Eiki Takahashi, Jumpei Yasuda, Shizukaze Matsuda, Ayumu Kawasaki, Masaki Watanabe, Kosuke Otani, Muneyoshi Okada, Masakazu Sekijima, Hideyuki Yamawaki, Nobuya Sasaki

**Affiliations:** 1 Laboratory of Laboratory Animal Science and Medicine, School of Veterinary Medicine, Kitasato University, Towada, Japan; 2 Laboratory of Toxicology, School of Veterinary Medicine, Kitasato University, Towada, Japan; 3 Department of Computer Science, Institute of Science Tokyo, Kanagawa, Japan; 4 Faculty of Veterinary Medicine, Hokkaido University, Sapporo, Japan; 5 Department of Biomedicine, Graduate School of Medical Sciences, Kyushu University, Fukuoka, Japan; 6 Department of Pharmacology, Wakayama Medical University, Wakayama, Japan; 7 Laboratory of Veterinary Pharmacology, School of Veterinary Medicine, Kitasato University, Towada, Japan; Tokyo Women's Medical University, JAPAN

## Abstract

Voltage-gated potassium (Kv) channels contribute to repolarization in excitable tissues such as nerves and cardiac muscle; consequently, they control the firing frequency and duration of action potential. Their dysfunction can thus cause neurological disorders and cardiac disorders with arrhythmias. The dysfunction of Kv11.3 is associated with bipolar disorder, but no reports have linked it to heart disease. Kv11.3-knocked out (KO) mice exhibit behavioral abnormalities, but they do not have cardiac abnormalities. Ischemia–reperfusion (I/R) experiments were performed on the hearts of Kv11.3 KO mice to determine whether they would differ from wild-type mice when exposed to stimuli that could induce sudden cardiac death. The mortality rates and infarct size of the Kv11.3 KO mice increased after cardiac I/R. The corrected QT interval was shortened in the wild-type mice after cardiac I/R, but it remained nearly unchanged in Kv11.3 KO mice with alterations in heart rate variability. These phenotypes could be reproduced by administering high-dose NS-1643, a Kv11.3 channel antagonist, after cardiac I/R. The infarct size had no significant difference in the *ex vivo* cardiac I/R experiment in contrast to the *in vivo* cardiac I/R experiment. Our study indicated that Kv11.3 protects the myocardium from I/R injury through neural pathways.

## Introduction

Potassium voltage-gated (Kv) channels are divided into 12 subfamilies based on sequence similarities: Kv1 to Kv12. Physiologically, they mainly contribute to repolarization in excitable tissues such as nerves and cardiac muscle; consequently, they control the firing frequency and duration of action potential [1]. Therefore, their dysfunction can cause various neurological disorders, such as episodic ataxia (*KCNA1*/Kv1.1), epilepsy and epileptic disorders (*KCNA2*/Kv1.2, *KCNB1*/Kv2.1, *KCNC1*/Kv3.1, *KCNQ2*/Kv7.2, and *KCNQ3*/Kv7.3) [2], and cardiac disorders with arrhythmias, such as atrial fibrillation, long QT syndrome (*KCNQ1*/Kv7.1 and *KCNH2*/Kv11.1) [3], and Brugada syndrome (*KCND3*/Kv4.3) [4].

Bipolar disorder is a psychiatric disorder characterized by alternating episodes of elevated mood “mania” and depressed mood “depression.” Although genetic studies have identified several candidate genes for this disorder, none of them are critical, and the disorder is considered polygenic [5]. Among Kv channels, *KCNQ2*, *KCNQ3*, and *KCNH7*/Kv11.3 have been identified as candidate genes for bipolar disorder [6–8]. Interestingly, all these Kv channels exhibit a resonance property, and they participate in membrane potential oscillations [9,10]. Epidemiological data indicate that the risk of sudden cardiac death in patients with bipolar disorder under 30 years of age is approximately 32 times higher than that of the general population [11]. The risk of sudden cardiac death is higher in patients with severe psychiatric disorders [12], but the risk ratio in patients with bipolar disorder is particularly high. In a disease model, disease-associated genes may directly influence cardiac function, or the pathophysiology of bipolar disorder may affect the heart; however, its mechanisms remain unclear.

According to the International Mouse Phenotyping Consortium (www.mousephenotype.org), knockout (KO) mice with Kv7.3 and Kv11.3 exhibit abnormal phenotypes in the open field test, while they have no abnormalities in echocardiography or electrocardiograms [13]. In the present study, we performed ischemia/reperfusion (I/R) experiments on the hearts of Kv11.3 KO mice to determine whether they would differ from wild-type mice when exposed to stimuli that could induce sudden cardiac death. Cardiac I/R induces myocardial injuries, including cardiomyocyte death and cardiac arrhythmia [14].

## Materials and methods

### Antibodies

**Fig 1:**
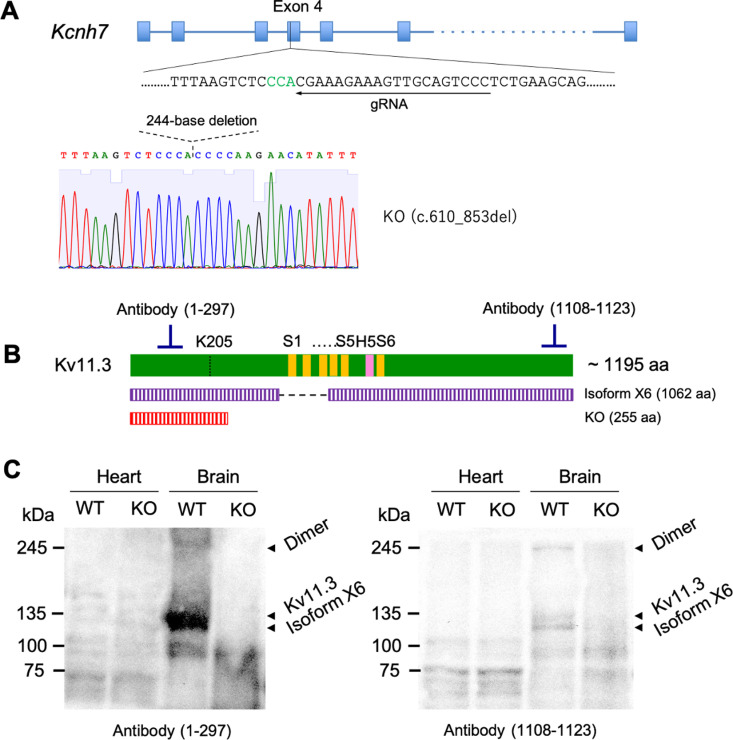
Generation of Kv11.3 KO mice. (A) The guide strand of the CRISPR/Cas9 system was designed to target the mouse Kcnh7 exon 4 (NCBI accession number NM_133207). DNA sequencing of the targeted region in knockout (KO) mice. (B) Schematic protein structure of Kv11.3. The mutant N-terminally truncated Kv11.3 proteins generated in KO mice lose their functional domains include a pore-forming domain. S1–S6, transmembrane helices. H5, pore-forming helix. (C) Western blotting for Kv11.3 in the whole heart and brain lysates. In KO brain, the bands of full-length Kv11.3 (including dimer) and Kv11.3 isoform X6 (NCBI accession number XP_017171420) were not detected. Original blots are presented in S4 Fig.

The following antibodies were used: rabbit polyclonal antibody against Kv11.3 C-terminal region (positions 1108–1123; GTX16686, GeneTex, Irvine, CA, USA); rabbit polyclonal antibody against Kv11.3 N-terminal region (positions 1–297; 13622–1-AP, Proteintech, Rosemont, IL, USA). The specificity of the anti-Kv11.3 antibody was confirmed by Western blotting in murine brains, where Kv11.3 expression has been experimentally verified (Fig 1C) [15].

### Animals

For this study, C57BL/6J mice were purchased from CLEA Japan (Tokyo, Japan). The animal housing facility was airconditioned, with temperature maintained at 22 ± 2°C and relative humidity at 40–60%. The mice were maintained under a 12-h light–dark cycle. A basal diet, CE-2 (CLEA Japan), and tap water were available *ad libitum*. The microbiological status of the study animals was monitored periodically in accordance with the guidelines of the Japanese Association of Laboratory Animal Facilities of Public and Private Universities. All research procedures were performed in accordance with the NIH Guide for the Care and Use of Laboratory Animals and approved by the President of Kitasato University based on the judgment by the Institutional Animal Care and Use Committee of Kitasato University (Approval ID: 19–161, 20–088, 23–071). All animal reporting was conducted in accordance with ARRIVE guidelines.

### Genome editing in mice

CRISPR/Cas9 genome editing was performed using pSpCas9(BB)-2A-Puro (PX459) V2.0 (#62988, Addgene) [16] – a plasmid that expresses a chimeric guide RNA (gRNA), a puromycin resistance gene, and a Cas9. The gRNA guiding sequence was designed as follows: 5’-GGGACTGCAACTTTCTTTCG-3’, which targets the mouse *Kcnh7* exon 4 (NCBI accession number: NM_133207). The gRNA-inserted PX459 V2.0 plasmid was delivered briefly into fertilized eggs from C57BL/6J mice by microinjection. After overnight culture, two-cell embryos were transferred into pseudopregnant female mice. Offspring carrying a KO mutation were identified and confirmed by DNA sequencing, and the mutant strains were maintained by backcrossing to C57BL/6J mice. Homozygous mutant mice were generated by crossing between more than N3-generation heterozygous mutant mice themselves and the offspring thus obtained were used for the experiments.

### Western blot analysis

Mice (weighing approximately 30 g) were euthanized by cervical dislocation. Fresh hearts and brains harvested from the mice were frozen, homogenized and lysed in RIPA buffer. Protein contents in the lysates were measured using a Precision Red Advanced Protein Assay reagent (Cytoskeleton, Denver, CO, USA). Lysates were boiled with 2% sodium dodecyl sulfate (SDS) and 5% 2-mercaptoethanol, electrophoresed in an SDS-polyacrylamide gel and blotted on polyvinylidene difluoride membranes. For blocking non-specific binding, the membranes were incubated in a Blocking One reagent (Nacalai Tesque, Kyoto, Japan) for 5 min at room temperature. The membranes were incubated with primary antibody (1:5000) for 1 h at room temperature. Thereafter, the membranes were incubated with secondary horseradish peroxidase-conjugated antibodies against rabbit IgG (1:10000, Cell Signaling Technology, Danvers, MA, USA) for 1 h at room temperature. After incubation with an ECL Prime detection reagent (Merck, Darmstadt, Germany), the blots were imaged using an Omega Lum C imaging system (Gel Co., San Francisco, CA, USA).

### *ex vivo* I/R model

Male and female mice older than 30 weeks were anesthetized with a mixture of secobarbital and butorphanol (SB; 60 and 5 mg/kg, i.p.). The heart was immediately isolated and perfused with normal-2-[4-(2-hydroxyethyl)-1-piperazinyl] ethanesulfonic acid HEPES-Tyrode solution (143 mM NaCl, 5.4 mM KCl, 0.33 mM NaH2PO4, 0.5 mM MgCl2, 5.5 mM glucose, 5 mM HEPES, 1.8 mM CaCl2, and 1N NaOH at pH 7.4) at constant pressure (80 cm H_2_O) via the aorta by using the Langendorff apparatus. Mice were euthanized by exsanguination under anesthesia. After 10-min perfusion, the hearts were exposed to 30-min global ischemia, followed by 2-h reperfusion. Hearts were excluded if normal beating was not resumed upon reperfusion.

### *in vivo* I/R model

**Fig 2:**
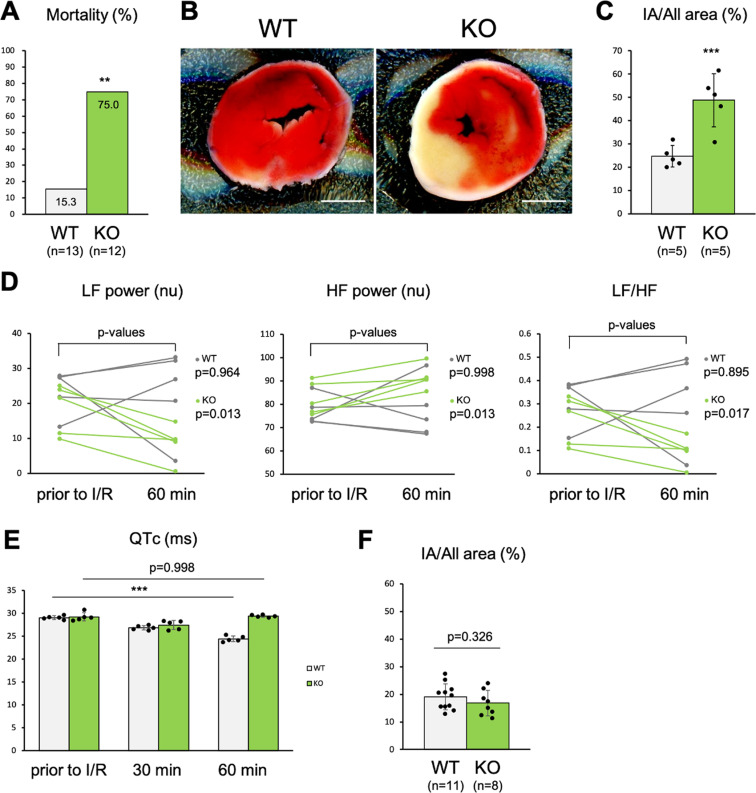
Kv11.3 deficiency in the mouse model of cardiac ischemia/reperfusion (I/R). (A) The mortality rate during 2-h reperfusion: wild-type mice (WT), n = 13 mice. Kv11.3 knockout mice (KO), n = 12 mice. (**B**) 2,3,5-triphenyltetrazolium chloride (TTC) staining of the hearts exposed to 1.5-h reperfusion. Scale bars, 2 mm. (C) The proportion of infarct area (IA) to all areas in the hearts exposed to 1.5-h reperfusion, n = 5 mice per genotype. (D, E) The heart rate variability frequency domain metrics and the corrected QT interval (QTc) before and after I/R. LF, low frequency. HF, high frequency. n = 5 mice per genotype. (F) The proportion of IA to all area in the Langendorff-perfused hearts. WT, n = 11 mice. KO, n = 8 mice. *P*-values, *** < 0.001, ** < 0.01, * < 0.05. Statistical significance was determined by Fisher’s exact test (A), unpaired Student’s t-test (C, F), paired Student’s t-test (D) and Tukey-Kramer test (E).

**Fig 3:**
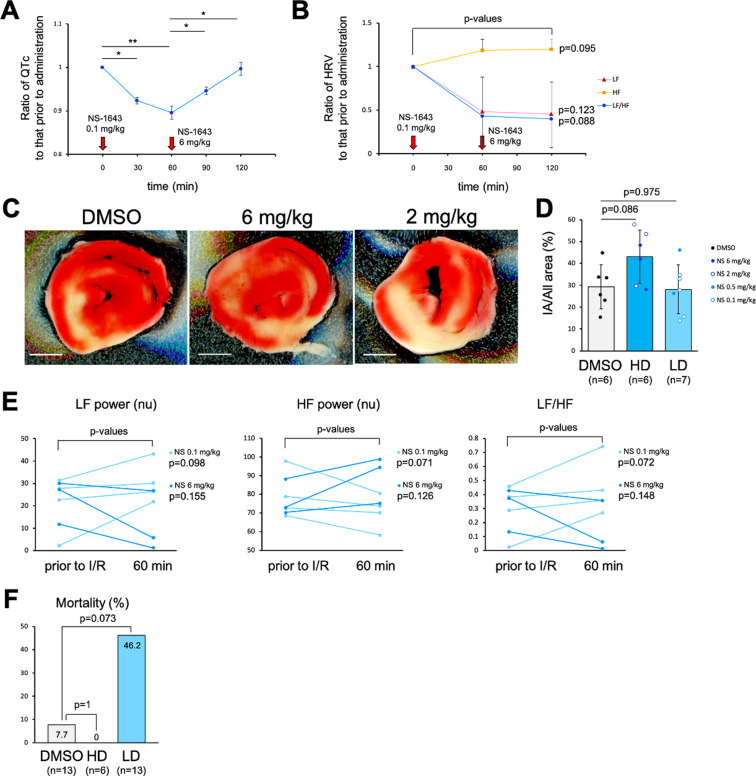
Effect of NS-1643 in a murine cardiac ischemia/reperfusion (I/R) model. (A, B) Ratio of the corrected QT interval (QTc) and the heart rate variability (HRV) frequency domain metrics in sham-operated wild-type mice treated with NS-1643. Arrows represent injections. LF, low frequency. HF, high frequency. n = 3 mice. (**C**) 2,3,5-triphenyltetrazolium chloride (TTC) staining of the hearts that were treated with high concentrations of NS-1643 and exposed to 2-h reperfusion. Scale bars, 2 mm. (D) The proportion of infarct area (IA) to all areas in the reperfused hearts treated with NS-1643. DMSO, dimethyl sulfoxide; HD, high-dose NS-1643 at 2 or 6 mg/kg; LD, low-dose NS-1643 at 0.1 or 0.5 mg/kg. DMSO, n = 6 mice. HD, n = 6 mice. LD, n = 7 mice. (E) Heart rate variability frequency domain metrics before and after I/R. LD (0.1 mg/kg), n = 4 mice. HD (6 mg/kg), n = 3 mice. (F) The mortality rate during 2-h reperfusion. DMSO, n = 13 mice. HD, n = 6 mice. LD, n = 13 mice. *P*-values, ** < 0.01, * < 0.05. Statistical significance was determined by paired Student’s *t*-tes*t* (A, B), Dunnett’s test (D), paired Student’s *t*-tes*t* (E), and Fisher’s exact test (F).


Male and female mice older than 30 weeks (weighing 30–40 g) were anesthetized with a mixture of medetomidine, midazolam, and butorphanol (MMB; 0.75, 4, and 5 mg/kg, respectively, i.p.) or SB before intubation and mechanical ventilation (0.8–1.5 cc, 90–125 breaths/min). MMB and SB were used for Fig 2A, and Figs 2B–E and 3, respectively. Following a bolus injection of 600 µ L saline, the thorax was surgically opened using a cautery and the pericardium and phrenic nerve were resected. Electrocardiogram (ECG) electrodes were connected to the left and right armpit and the left groin, and ECGs were recorded using the Power Lab system (ADI Instrument, New South Wales, Australia). Records were excluded if ECGs were missing or unreadable. Left anterior descending artery (LAD) was occluded using a hanging weight system [17]; after 30-min LAD occlusion, the hearts were reperfused for 1.5–2 h. After reperfusion, mice were euthanized by cervical dislocation. Mice were excluded if blood flow did not immediately return to the LAD upon reperfusion or if they died within 1.5 h of reperfusion. The sham-operated control mice underwent thoracotomy without LAD occlusion. NS-1643 (0.1–6 mg/kg) was administered intraperitoneally just before reperfusion and the ECG QT and RR intervals were measured manually and blindly on the Lead II recording. Heart rate variability (HRV) analysis was performed using the LabChart pro (ADI Instrument). Low frequency (LF) and high frequency (HF) powers (ms^2^) were converted into normalized unit (nu) using the following formula:

$$LF\;power\;[nu]=\frac{100\times LF\;power\;[{ms}^{2}]}{total\;power\;\left[{ms}^{2}\right]-very\;low\;frequency\;power\;[{ms}^{2}]}$$

$$HF\;power\;[nu]=\frac{100\times HF\;power\;[{ms}^{2}]}{total\;power\;\left[{ms}^{2}\right]-very\;low\;frequency\;power\;[{ms}^{2}]}$$

The QT interval was measured from the beginning of the QRS complex to the point at which the T wave reached the baseline. The corrected QT interval (QTc) was then calculated using Mitchell’s formula [18]:


$$\text{QTc}\;\text{=}\;\text{QT/}{\left(\text{RR/100}\right)}^{\text{1/2}}$$

### TTC staining

Myocardial infarct size was assessed after 2,3,5-triphenyltetrazolium chloride (TTC) staining. Precisely 5 min before the cessation of *in vivo* reperfusion, a one-third dose of SB was administered intraperitoneally. After reperfusion, the inferior vena cava was surgically exposed and incised, and 5 mL saline was perfused via the orbital sinus. Mice were euthanized by exsanguination under anesthesia. The heart was excised, washed in ice-cold saline, embedded in a fish gelatin (Gelatin Fine Mesh F, Jeleaf, Yasu, Japan) and agarose mixture (gelatin, 1%; agarose, 0.4%) with a low melting temperature, and then placed at 4°C for 30 min. Thereafter, 1-mm thick slices of the heart were obtained using a heart matrix and then incubated in 1% TTC solution at 37°C for 10 min, placed in 10% formaldehyde for 5 days, and imaged using a standard commercial scanner. The infarcted area and the cardiac parenchymal area were measured blindly using Image J (National Institutes of Health, Bethesda, MD, USA).

### *in silico* molecular docking

The 3D structure information of human Kv channel proteins was obtained from the Protein Data Bank (PDB), and their predicted 3D structures were obtained from the AlphaFold2 Database (IDs shown in S1 Table). The 3D structure of NS-1643 was obtained from PubChem (CID: 10177784), and the topological domain information was acquired according to UniProt. Molecular docking and molecular dynamic (MD) simulations were performed using the Schrödinger Small Molecule Discovery suite version 2021–4 (Schrödinger, New York, NY, USA) according to the previous report [19]. To determine the binding sites of NS-1643 in the proteins, the amino acid sequence was aligned with the Crustal W method by using MEGA11 [20]. Guo *et al*. reported that NS-1643 interacts with Kv11.1 near L529 and around the S4 helix, which is one of six transmembrane helices of Kv proteins [21], and the alignment analysis showed that R528 is conserved in the Kv families. Therefore, the box’s centroid was adjusted according to the R528 equivalent of each one of these families. As the structure predicted by alphafold2 includes non-folded degenerate regions that are not seen in the structures from PDB, the following amino acids were excluded: Kv11.1, 134^th^–369^th^ and 871^st^–1159^th^; Kv11.2, 149^th^–216^th^ and 721^st^–958^th^; Kv11.3, 1^st^–266^th^ and 780^th^–1096^th^; Kv4.3, 511^th^–636^th^; Kv4.2, 509^th^–630^th^; and Kv7.1, 397^th^–500^th^. The active site was defined by a 30 Å × 30 Å × 30 Å grid box. The OPLS4 force field was used for structure minimization and grid-box generation [22]. NS-1643 was docked into the binding site of Kv channel proteins by using Glide software (Schrödinger) with a standard precision-scoring function [23]. A docking score of up to −7.0 kcal/mol and an e-model score of up to −50 kcal/mol were used as standard thresholds [24].

### *in silico* MD simulations

MD simulation of Kv channel proteins was performed with and without NS-1643 using GPU-accelerated Desmond software (D.E. Shaw Research, New York, NY, USA) [25]. Each protein–ligand complex was built using the best Glide docking pose at the initial starting point, which was then explicitly solved with the TIP3P water model, 0.15 M KCl aqueous salt solution, and a POPC lipid bilayer model. The full-atom simulation was run for 50 ns using OPLS4 force field energy minimization and a 10-Å orthorhombic volume; default settings were used for all other parameters. During the 50 ns simulation, 2500 trajectories were recorded. To analyze these MD simulations, the Simulation Interactions Diagram tool was used to perform an interaction analysis between Kv channel proteins and NS-1643 and to calculate root mean square fluctuation (RMSF) values. The RMSF for residue i is as follows:


$$RMSF_{i}=\sqrt{\frac{1}{T}\sum_{t=1}^{T}<}{\left(r_{i}^{\prime }(t)-r_{i}\left(t_{0}\right)\right)}^{2}>$$

where T is the trajectory time over which the RMSF is calculated; $t_{0}$ is the reference time; $r_{i}$ is the position of residue i; $r_{i}^{\prime }$ is the position of atoms in residue i after superposition on the reference; and the angle brackets indicate that the average of the square distance is obtained by the selection of atoms in the residue. The Desmond trajectory clustering tool was used to obtain representative poses of the Kv channel protein–NS-1643 complex. In the trajectory clustering, the backbone atom was set for the RMSD matrix, and the analysis was performed through the affinity propagation clustering method. The structure with the largest number of neighbors in the clustering was used for docking score calculation as the representative structure. The calculation was obtained through the Glide One Step Docking function.

### Statistical analysis

Data are expressed as the mean ± standard deviation. Statistical analyses were carried out using the JMP pro software (version 17.0.0). Statistical significance was determined by the Student’s *t*-test, Fisher’s exact test, Dunnett’s test and Tukey-Kramer test. All tests were two sided. A *p*-value of < 0.05 was considered statistically significant.

## Results

### Kv11.3 deficiency aggravates myocardial I/R injury

Kv11.3 KO mice were generated by using the CRISPR/Cas9 system (Fig 1). They were subjected to the LAD occlusion for 30 min and reperfusion for 2h. The results revealed that some mice developed pulmonary congestion, which led to respiratory arrest and death at approximately 15 min before the endpoint. The mortality rate was significantly higher in the Kv11.3 KO mice than in the wild-type mice (75.0% vs. 15.3%, respectively, *p* < 0.01; Fig 2A). The infarct sizes of Kv11.3 KO mice were significantly larger than those of the wild-type mice (Figs 2B and 2C). Therefore, Kv11.3 potentially provided protection against myocardial I/R injury.

The HRV frequency domain metrics of the wild-type mice, including LF power, HF power, and ratio of LF to HF power (LF/HF), remained almost unchanged by cardiac I/R, whereas the LF/HF of Kv11.3 KO mice was decreased as a result of a decrease in the LF power and an increase in the HF power (Fig 2D). The QTc of the wild-type mice was shortened by 15.9% within 60 min after cardiac I/R; by comparison, the QTc of the Kv11.3 KO mice remained almost unchanged (Fig 2E).

Western blot analysis revealed that Kv11.3 expression was detected in the brain but not in the heart (Fig 1C). Furthermore, we performed similar I/R experiments by using a Langendorff-perfused heart. We found no significant differences in the infarct sizes between the genotypes (Fig 2F).

### Pharmacological blockade of Kv11.3 aggravates myocardial I/R injury

In the mouse model of cardiac I/R, we evaluated the efficacy of NS-1643, which can modify the Kv11 family channel activity. Approximately 10 µ M NS-1643 activates Kv11.1 [26], *KCNH6*/Kv11.2 [27], and Kv11.3 [28] channels; at a higher concentration of 20 µ M, NS-1643 functions as an agonist of Kv11.2 and inversely exerts partial antagonistic effects on Kv11.1 and Kv11.3 [28,29]. In sham-operated wild-type mice, QTc shortening was induced by intraperitoneally administering low-dose NS-1643 (0.1 mg/kg of body weight; Fig 3A). QTc was restored by subsequently administering high-dose NS-1643 (6 mg/kg of body weight; Fig 3A). The effect of NS-1643 on QTc is thought to be exerted through myocardial Kv11.1, which contributes to repolarization [30]. Such adverse effects in an NS-1643 concentration-dependent manner were not observed in HRV frequency domain metrics (Fig 3B). Consistent with the results in Kv11.3 KO mice, high-dose NS-1643 (2 or 6 mg/kg), which might function as a Kv11.3 channel antagonist, tended to increase the infarct size and decrease the LF/HF after cardiac I/R (Figs 3C–E). Inversely, low-dose NS-1643 (0.1 or 0.5 mg/kg), which might behave as a Kv11.3 channel agonist, tended to increase the LF/HF as a result of an increase in the LF power and a decrease in the HF power (Fig 3E). However, unexpectedly, low-dose NS-1643 increased mortality after cardiac I/R (Fig 3F).

### Does low-dose NS-1643 activate the Kv4.2/4.3 channel?

Low-dose NS-1643 did not increase the infarct size after cardiac I/R (Fig 3D). Therefore, the low-dose NS-1643-induced death after cardiac I/R was unlikely caused by myocardial I/R injury. Besides the Kv11 family, the channel activities of Kv4.3 and Kv7.1 are affected by NS-1643 [26]. Kv4.3 and Kv7.1 contribute to the fast transient outward potassium current (I_to,f_) and delayed rectifier potassium current (I_Ks_) in cardiomyocytes, respectively [30]. Interestingly, the gain-of-function mutations associated with Brugada syndrome have been identified in *KCND3* and *KCND2* encoding Kv4.2, which constitutes a Kv channel tetramer, along with Kv4.3 *in vivo* [4,31,32]. Brugada syndrome is an inherited arrhythmogenic disorder related to the risk of recurrent ventricular fibrillation and sudden cardiac death [33]. High-dose NS-1643 (30 µ M) inhibits Kv4.3 and Kv7.1 channels [26]. The effects of low-dose NS-1643 are unknown. Similar to Kv11.1 and Kv11.3, if Kv4.2 and Kv4.3 channels exert adverse activities in an NS-1643 concentration-dependent manner, their activation may have caused the low-dose NS-1643-induced death after cardiac I/R.

**Fig 4:**
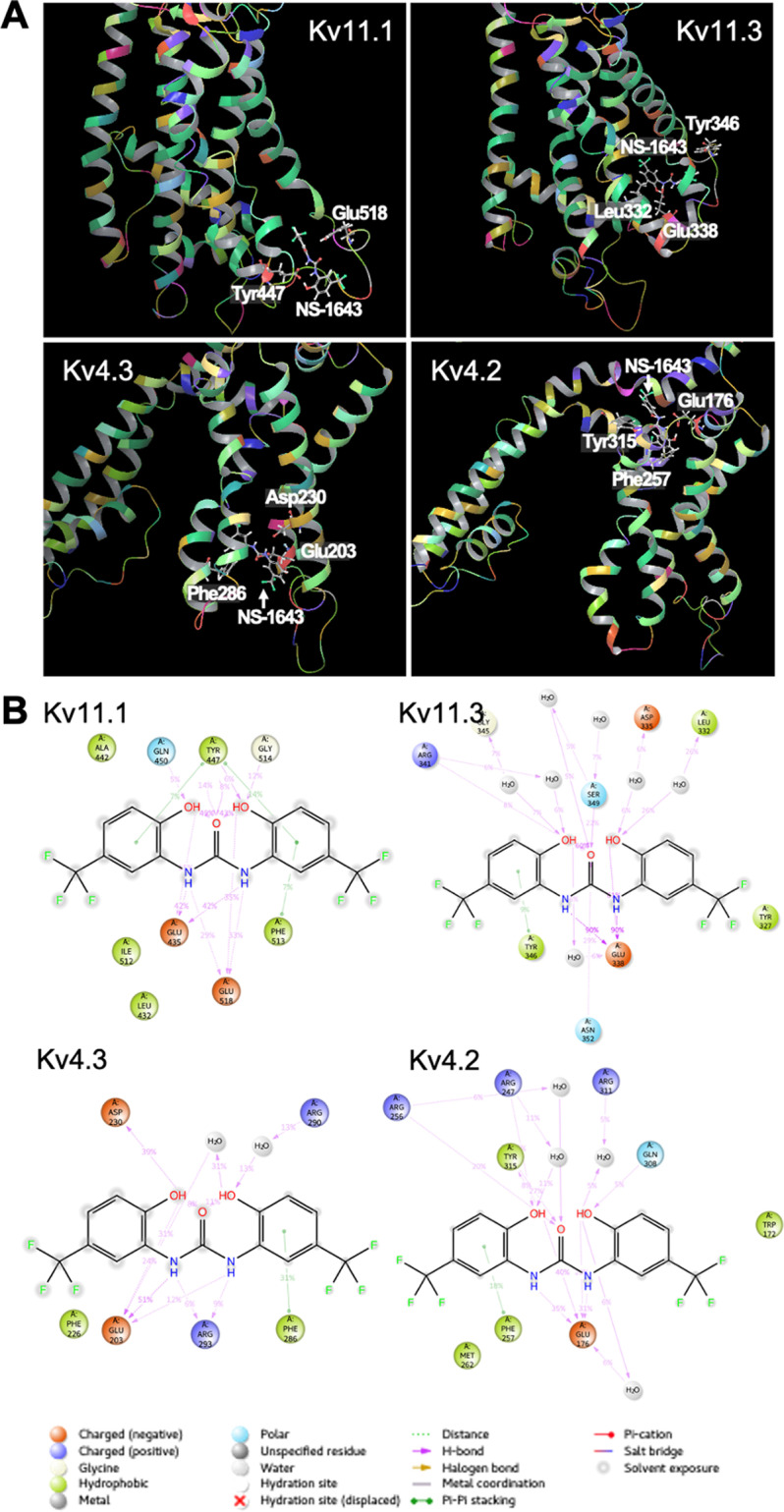
The predicted interactions of the Kv channel proteins (Kv11.1, Kv11.3, Kv4.3, and Kv4.2) with NS-1643. (A) Theoretical binding poses of Kv channel proteins with NS-1643 obtained by molecular dynamic (MD) trajectory clustering. The most frequent structure that occurred during the simulation is shown. The protein is illustrated in ribbon representation with the representative binding sites in ball and stick representation. (B) Schematic representation of the interactions between Kv channel proteins and NS-1643 that occur over more than 5.0% of the 50.0 ns simulation time are depicted as arrows.

Molecular docking and MD simulations of Kv channel proteins with NS-1643 were performed to support this hypothesis. The glide docking and e-model scores are shown in S1 Table. Only Kv11.1 and Kv11.3 had favorable initial docking scores of less than −7.0 kcal/mol; by comparison, all docked poses except for Kv7.1 demonstrated generally good e-model scores of up to approximately −50 kcal/mol. The MD simulations of these Kv channel protein/NS-1643 complexes and Kv channel protein alone were conducted to observe their dynamic changes over time. The RMSF values of each amino acid in the protein chain during the 50 nsec simulation is shown in S1 Fig. The RMSF indicates the mobilization of each amino acid residue from its initial structure at the start of MD simulation (i.e., local changes along the protein chain). The mobilities of Kv11.2 and Kv4.3 were greater throughout the protein chains when bound to NS-1643. In Kv11.1, Kv11.3, and Kv4.2, the increased mobility caused by the binding of NS-1643 was partially detected, especially around the transmembrane core of Kv channels, including S1–S6 transmembrane helices. Conversely, the binding of NS-1643 reduced mobility around the transmembrane core of Kv7.1. The interactions of the Kv channel proteins with NS-1643 are illustrated in Fig 4 and S2 Fig. NS-1643 was docked into a binding pocket formed by the extracellular or cytoplasmic domains of the Kv channel proteins except Kv7.1. NS-1643 was held in place by the binding of a negatively charged residue (Glu or Asp; in orange) to the secondary amine in the NS-1643. It was also maintained by the π-π stacking of the aromatic rings with the hydrophobic residues Tyr and/or Phe (in light green). Remarkably, Kv11.2 was connected to NS-1643 by the binding of the polar residue Thr (in light blue) and by the π-π stacking of the polar residue His. In Kv4.2, Glu frequently interacted with NS-1643 in the first half of the simulation; however, in Kv11.1, Kv11.3, and Kv4.3, this interaction occurred throughout the simulation (the secondary amine-binding residue in Kv11.1 switched from Glu518 to Glu435; S3 Fig). In summary, our simulations indicate that the binding modes with NS-1643 of Kv4.2 and Kv4.3 resembled those of Kv11.1 and Kv11.3.

## Discussion

Kv11.3 belongs to a Kv channel family that includes Kv11.1, which has been well studied in cardiomyocytes; its RNA expression shows tissue specificity for the cerebral cortex [34]. In the present study, we performed cardiac I/R experiments on Kv11.3 KO mice to determine whether they differed from wild-type mice when exposed to stimuli that could induce sudden cardiac death. Consistent with previous reports, our study did not find cardiac abnormalities in Kv11.3 KO mice under regular growth conditions. However, Kv11.3 deficiency significantly increased the mortality rate and infarct size after cardiac I/R. This finding suggested that Kv11.3 protects against myocardial I/R injury. The infarct size had no significant difference in *ex vivo* cardiac IR experiments in contrast to the *in vivo* cardiac IR experiments. Therefore, these results indicated that the effect of Kv11.3 is mediated via the nervous system. Moreover, our experiments revealed that the QTc of wild-type mice was shortened by cardiac I/R, whereas the QTc of Kv11.3 KO mice remained nearly unchanged with a decrease in the LF/HF. A high concentration of NS-1643, which can behave as a Kv11.3 channel antagonist, increased infarct size and decreased the LF/HF after cardiac I/R. QT interval shortening indicates a shortening of action potential duration (APD). The heart is innervated by the sympathetic and vagus nerves, where sympathetic activation promotes myocardial repolarization and shortens the APD [35]. Both sympathetic and vagal activations are elicited by myocardial ischemia [36]. LF and HF powers are influenced by autonomic nervous system activity [37]. Numerous studies have provided evidence describing the autonomic mechanism of protecting the heart against I/R injury [36]. For example, vagus nerve stimulation reduces myocardial I/R injury. Kv11.3 expression is also detected in hypothalamus, which is a center of the sympathetic nervous system, and the dorsal motor nucleus of the vagus [34,38]. Therefore, in the hearts of wild-type mice after IR, autonomic nervous modulation mediated by the central nervous system likely protects the myocardium. Conversely, this acute response appears to be impaired in the hearts of Kv11.3 KO mice. The sensitivity of the heart to sympathetic and parasympathetic input decreases with age [39–41], indicating that the responsiveness of the heart to stress diminishes as people age. This finding is consistent with the fact that the risk of sudden cardiac death increases with age [42].

QT interval prolongation after cardiac I/R events has been clinically observed in humans [43]. However, few studies have reported the QT interval in the mouse models of cardiac I/R. Our results differed from those of human studies possibly because components of ventricular repolarization currents vary between human and mice [44]. The rapid component of delayed rectifier potassium current (I_Kr_) and I_Ks_ are crucial for repolarization in humans, but these factors are negligible in mice. Conversely, the ultra-rapid delayed rectifier potassium current (I_Kur_) is essential for repolarization in mice, but it is undetectable in the humans. In addition, Kv11.1, which is a major ion channel that conducts I_Kr_ in human cardiomyocytes [30], is sensitive to oxidative stress [45]. Therefore, an I/R-induced increase in reactive oxygen species possibly decreases I_Kr_, with a resultant QT interval prolongation [46]. In mice, this inhibitory effect should be negligible.

To investigate whether the activation of the Kv11.3 channel can alleviate I/R injury more effectively, we tested low-dose NS-1643, which can behave as a Kv11.3 channel agonist. However, unexpectedly, low-dose NS-1643 administration increased the mortality after cardiac I/R. As low-dose NS-1643 administration did not increase the infarct size after cardiac I/R, this lethality did not seem to have been caused by the myocardial I/R injury. Therefore, in this study, we focused on the interactions of NS-1643 with Kv4.2/4.3 and Kv7.1, whose channel activation is related to Brugada syndrome [4,31,32], which can lead to recurrent ventricular fibrillation and sudden cardiac death [33]. Since high-dose NS-1643 inhibits Kv4.3 and Kv7.1 channels [26], low-dose NS-1643 likely activates these channels inversely, similarly to its effect on Kv11.1 and Kv11.3. If so, low-dose NS-1643 causes lethal arrhythmia through the activation of the Kv4.2/4.3 or Kv7.1 channel. To support this hypothesis, we performed molecular docking and MD simulations of Kv channel proteins with NS-1643. In summary, the simulations demonstrated the similarities in NS-1643-binding modes among the following Kv channel proteins: Kv11.1, Kv11.3, Kv4.2, and Kv4.3. Therefore, in the presence of NS-1643, the Kv4.2/4.3 channel (not the Kv7.1 channel) is expected to behave similarly to Kv11.1 and Kv11.3 channels. The RMSF analysis indicated that the mobility of the transmembrane core, including the voltage sensor domain and the pore-forming domain, was greater after NS-1643 binding except with Kv7.1. NS-1643 activates Kv11 channels mainly by increasing the voltage sensitivity of activation [28]. However, how does the voltage sensor domain of Kv channels sense the membrane potential change? A consensus model indicates that the transmembrane helices in the voltage sensor domain are movable, and the charged amino acid residues in these helices conformationally interact with one another [47]. The mechanism of the excitatory action of NS-1643 may involve facilitating these interactions by increasing the mobility of the transmembrane core.

The major limitation of our study is the small sample size for the NS-1643 administration experiments. Furthermore, the target of NS-1643 is not specific to Kv11.3. The effects of NS-1643 in I/R model could be a result of the combined actions of Kv channels, including Kv11.1, which is expressed in both the brain and the heart. Finally, MD simulation with a tetramer constituting the entire channel is impossible because the number of molecules is too large. As such, longer term and larger graphics processing units are needed to perform such an MD simulation. However, a monomer’s evaluation is considered sufficient when a compound such as NS-1643 binds to a monomer in a multimer.

## Conclusion

In summary, we demonstrated that Kv11.3 protected the myocardium from I/R injury, and this protective effect might result from efferent autonomic modulation. Therefore, this function of Kv11.3 might explain why patients with bipolar disorder had a high risk of sudden cardiac death. Moreover, we presented novel modes of NS-1643 binding to Kv channels in which low-dose NS-1643 could activate the Kv4.2/4.3 channel.

## Supporting information

S1 Table Theoretical docking score. The binding energy between the Kv channel proteins and NS-1643, estimated by molecular docking, is shown. The protein structures (ID:AF-) have been predicted by AlphaFold2, and the remaining three protein structures have been obtained experimentally and deposited in the Protein Data Bank (PDB). The docking score indicates the binding energy; a score below −7.0 kcal/mol is often used as an indicator of pharmacological activity. The Glide e-Model indicates likelihood, and a score less than 50 kcal/mol is considered realistic. The docking score was calculated using the initial Kv channel protein structure wherein NS-1643 is denoted as the “initial,” whereas the score calculated by re-docking with the most frequent pose during molecular dynamic simulation is denoted as the “induced,” value.
(XLSX)

S1 Fig Root mean square fluctuation (RMSF) values for the protein chain. The blue dashed line and the red line were obtained from the trajectory analysis of the molecular dynamic simulations of the Kv channel protein and the Kv channel protein–NS-1643 complex, respectively. The green and yellow rectangles schematically represent the protein structures along with amino acid residues. S1–S6, transmembrane helices. H5, pore-forming helix.
(PNG)

S2 Fig The predicted interactions of the Kv channel proteins (Kv11.2 and Kv7.1) with NS-1643. (**A**) The theoretical binding poses of Kv channel proteins with NS-1643 obtained by molecular dynamic trajectory clustering. The most frequent structure generated during the simulation is shown. The protein is illustrated in ribbon representation, with the representative binding sites in ball and stick representation. (**B**) Schematic of the interactions between the Kv channel proteins and NS-1643 that occur over more than 5.0% of the 50.0 ns simulation time and are depicted as arrows.
(PNG)

S3 Fig A timeline representation of the predicted interactions and contacts between Kv channel proteins and NS-1643. The top panel shows the total number of specific contacts (H-bonds, Hydrophobic, Ionic, Water bridges), and the bottom panel present residues that interact with the NS-1643.
(PNG)

S4 Fig Uncropped blots of Fig 1C. (PNG)
