# Supplementary figures and images for: Protective function of the voltage-gated potassium channel Kv11.3 in a mouse model of cardiac ischemia/reperfusion injury
Source: PLoS One. 2025 May 7;20(5):e0323428. doi: 10.1371/journal.pone.0323428 (PMC12058134; doi:10.1371/journal.pone.0323428)

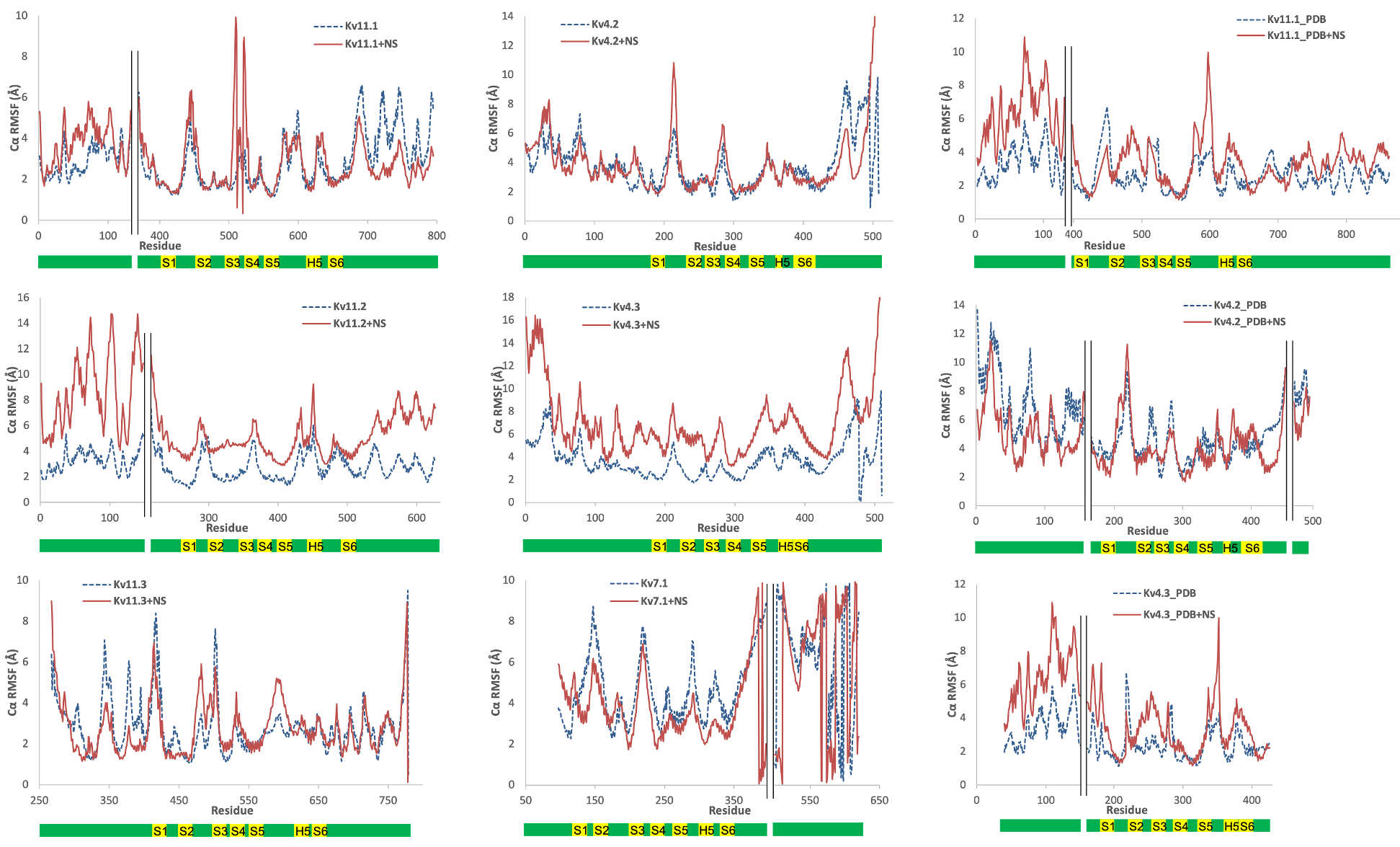

Supplement: S1 Fig — The blue dashed line and the red line were obtained from the trajectory analysis of the molecular dynamic simulations of the Kv channel protein and the Kv channel protein–NS-1643 complex, respectively. The green and yellow rectangles schematically represent the protein structures along with amino acid residues. S1–S6, transmembrane helices. H5, pore-forming helix. (PNG) [file pone.0323428.s002.png]

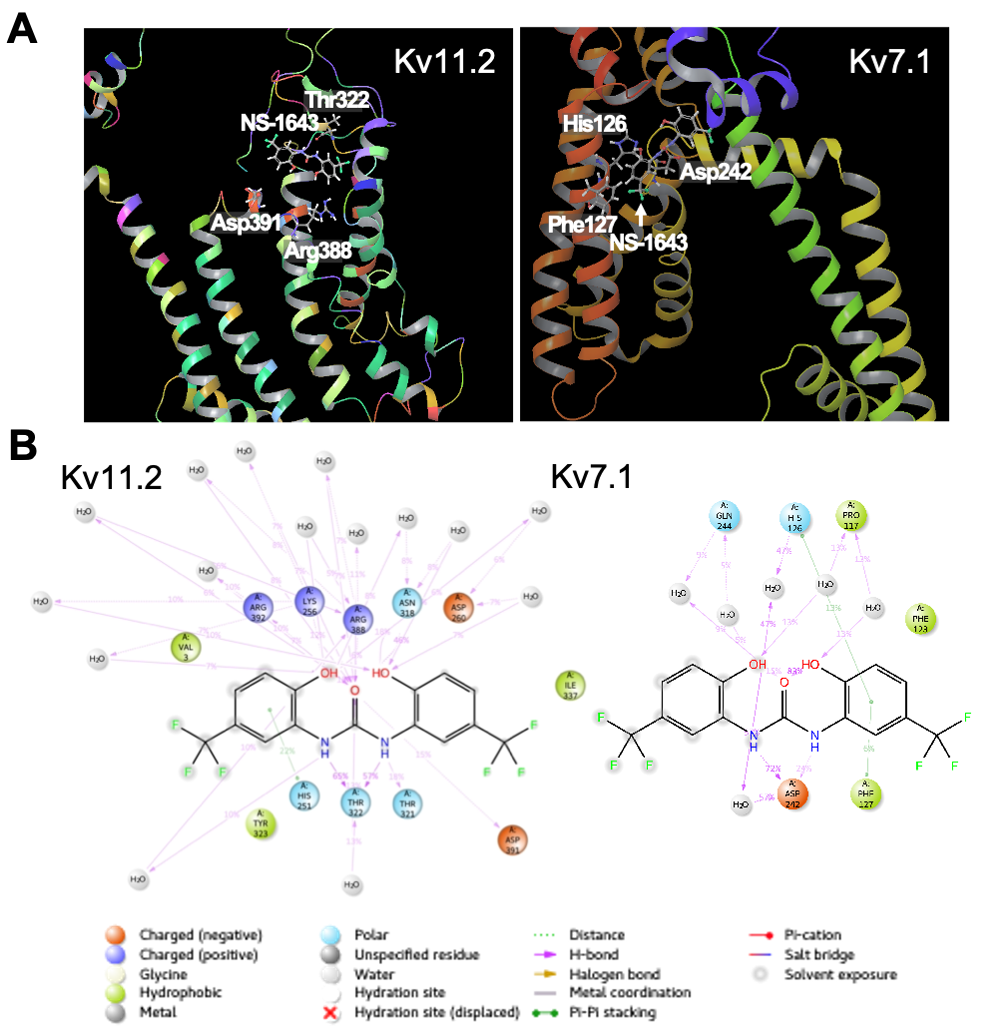

Supplement: S2 Fig — (A) The theoretical binding poses of Kv channel proteins with NS-1643 obtained by molecular dynamic trajectory clustering. The most frequent structure generated during the simulation is shown. The protein is illustrated in ribbon representation, with the representative binding sites in ball and stick representation. (B) Schematic of the interactions between the Kv channel proteins and NS-1643 that occur over more than 5.0% of the 50.0 ns simulation time and are depicted as arrows. (PNG) [file pone.0323428.s003.png]

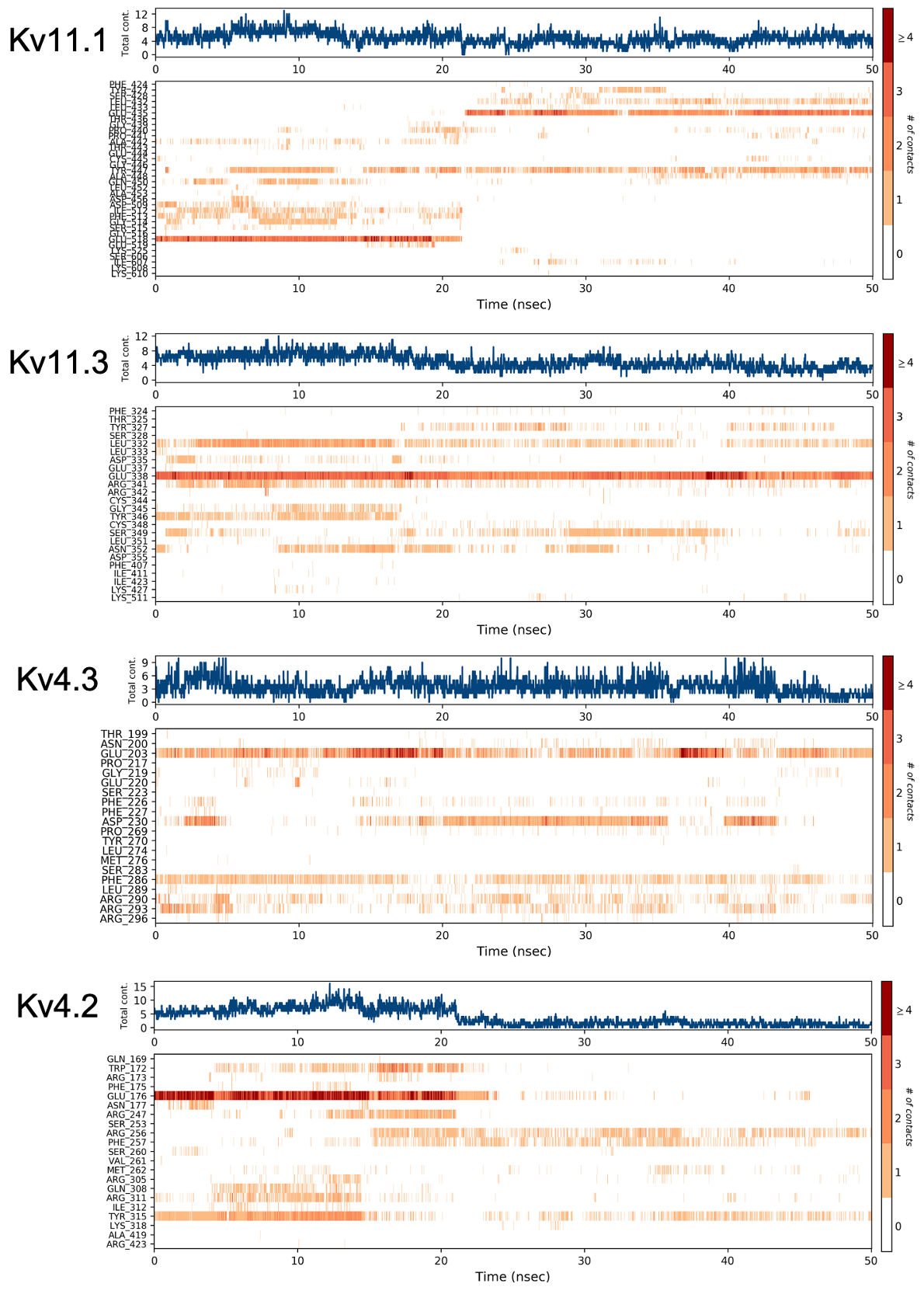

Supplement: S3 Fig — The top panel shows the total number of specific contacts (H-bonds, Hydrophobic, Ionic, Water bridges), and the bottom panel present residues that interact with the NS-1643. (PNG) [file pone.0323428.s004.png]

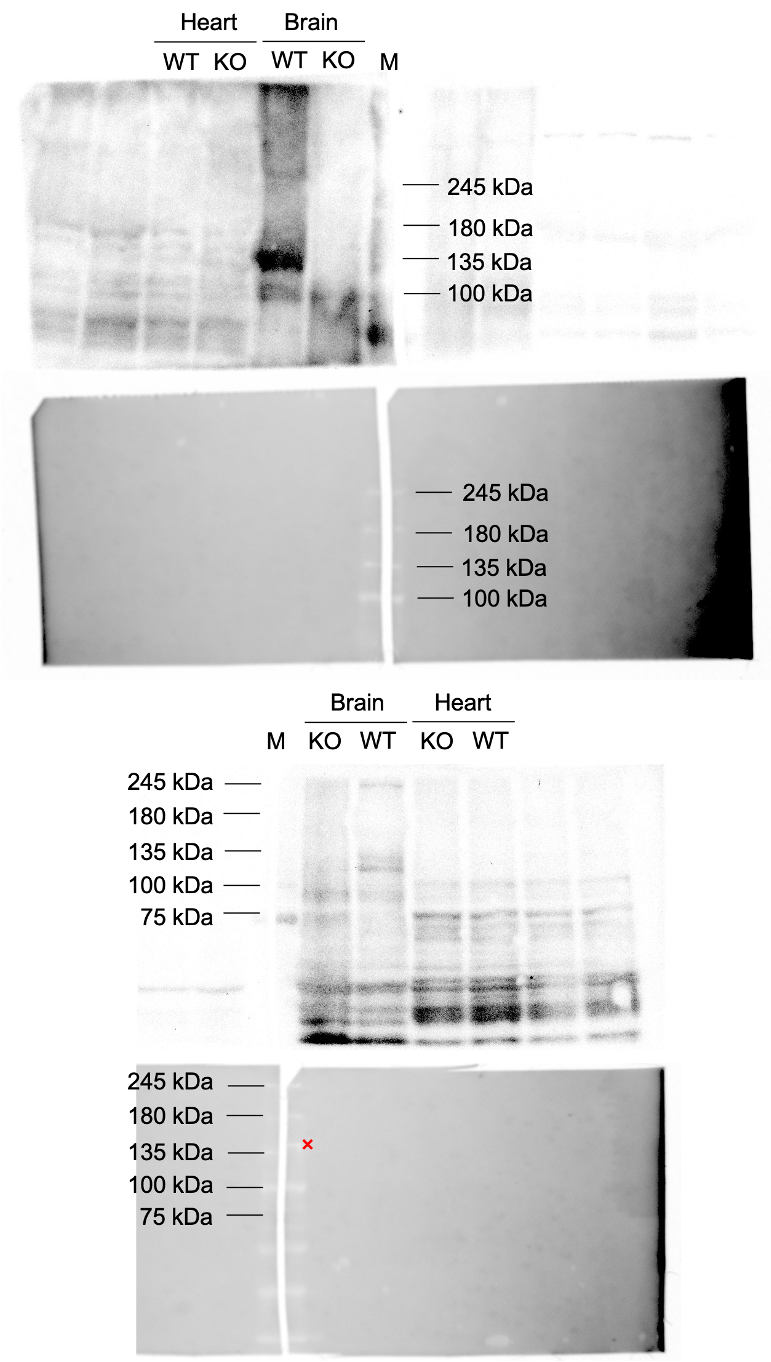

Supplement: S4 Fig — (PNG) [file pone.0323428.s005.png]
